# How do you target cognitive training? Bridging the gap between standard and technological rehabilitation of cognitive domains

**DOI:** 10.3389/fpsyg.2024.1497642

**Published:** 2024-11-07

**Authors:** Marina Maffoni, Antonia Pierobon, Daniela Mancini, Annalisa Magnani, Valeria Torlaschi, Cira Fundarò

**Affiliations:** ^1^Psychology Unit, Istituti Clinici Scientifici Maugeri IRCCS, Montescano Institute, Montescano, Italy; ^2^Neurophysiopatology Unit, Istituti Clinici Scientifici Maugeri IRCCS, Montescano Institute, Montescano, Italy

**Keywords:** rehabilitation, dementia, cognitive impairment, cognitive training, paper-pencil training, technological training

## 1 Introduction

Demographic forecasts suggest an increase of up to 3.1 billion people aged over 60 by the year 2100 (United Nations, 2017). Consequently, age-related issues—in particular cognitive decline—are increasing in frequency and significance. Due to the lack of effective drug therapies to cure cognitive disorders, addressing the socio-economic effects and risk factors of dementia-related disorders is an urgent and threatening global imperative (Jessen et al., 2023; Alzheimer's Disease Facts and Figures, 2024). The matter is further complicated by the fact that cognitive disorders can have different etiologies, severities, progressions and symptoms (Pérez Palmer et al., 2022). For instance, there are cases like Subjective Cognitive Decline (SCD)—that is a subclinical cognitive impairment—in which the symptoms could serve as a canary in a coal mine giving an early warning of future dementia (Röhr et al., 2020; Ribaldi et al., 2022). Thus, promoting early diagnosis and detection, as well as preventive, supportive and persistent lifestyle treatments is crucial to maximize results in counteracting and slowing cognitive decline (United Nations, 2017; United Nations, Department of Economic and Social Affairs, Population Division, 2024; Alzheimer's Disease Facts and Figures, 2024).

## 2 The relevance of cognitive training

**Figure 1:**
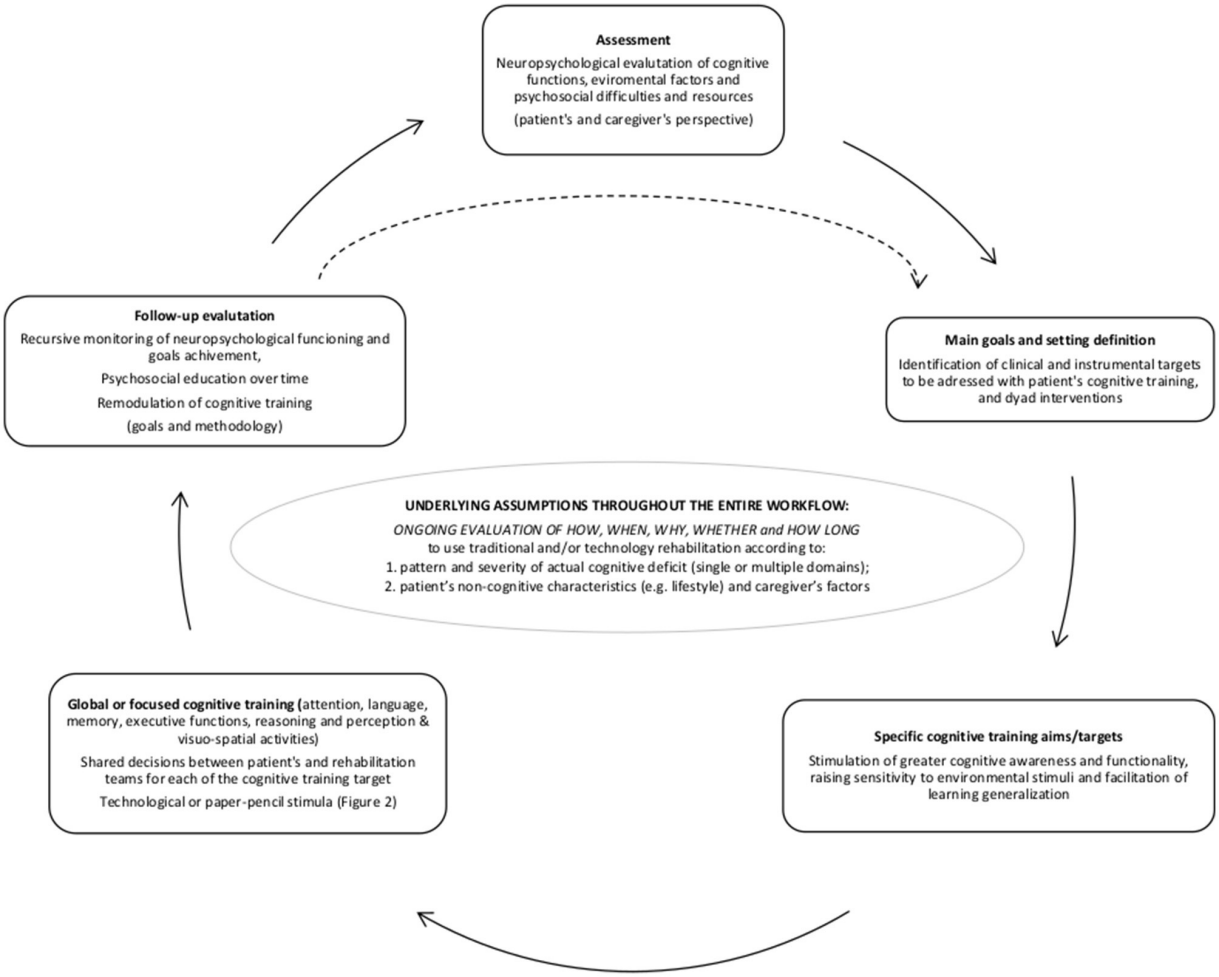
Patient-centered cognitive rehabilitation workflow.

Despite heterogeneity, non-pharmacological interventions are promising to prevent and take care of people with cognitive decline of varying severity and origin (Shimada et al., 2018; Yao et al., 2020; Li et al., 2023). Among them, cognitive training (CT) has gained attention due to the growing amount of literature describing its role in counteracting the cognitive deterioration process and improving patient's quality of life (Kudlicka et al., 2019; Ren et al., 2024). In this regard, promising preliminary evidence suggests the efficacy of brief screening tools integrated into daily clinical practice (Maffoni et al., 2022) as the early diagnosis is a prerequisite for the implementation of interventions, such as CT, that promote maintenance of existing cognitive resources in the prodromal phase and for slowing the rate of possible decline (Smart et al., 2017; Bernini et al., 2023a). To date, CT interventions are to be today considered a tailored strategy based on various kinds of tasks and exercises. These activities help people with different types of cognitive impairment to maximize and strengthen the residual cognitive resources, in an effort to both slow the decline and, mainly, to manage the daily challenges linked to the clinical condition (Mondini et al., 2016; Kudlicka et al., 2019). To accomplish that, the workflow for proposing and conducting effective training should be ongoing and tailored to the patient's rehabilitative needs and characteristics (Zampolini et al., 2022) (Figure 1).

## 3 Discussion: paper-pencil vs. technological cognitive rehabilitation: which ones?

If it is true that knowledge stands on the shoulders of giants, a consensus on *how, when, why*, and *whether* passing from standard cognitive rehabilitation to technological one is not only urgent but imperative for the scientific and clinical community. Indeed, CT should benefit from the use of technology, in line with the requests of the present and future healthcare scenario, and, in the meantime, take advantage of things learned by decades of experience with standard approaches.

Specifically, technology-based CT does not mean only adopting computers, but rather using a growing variety of technologies evolving over time (e.g., interactive video gaming, app, immersive or non-immersive software) and devices (e.g. smartphone, tablet, specific workstation) (Ge et al., 2018; Irazoki et al., 2020; Wilson et al., 2022).

### 3.1 Differences of standard and technological approaches

In the current healthcare landscape, the standard paper-pencil rehabilitation approach is giving the floor to new interventions derived from technology and telemedicine (Burton and O'Connell, 2018; Ge et al., 2018; Bernini et al., 2023a,b). Manifold differences may be unveiled concerning the paper-pencil approach.

Firstly, trainings with computers or tablets have gained attention as they may take advantage of more *catchy appealing stimuli*, and more *monitorable approach* (Hu et al., 2021; Wilson et al., 2022), which enhance *motivation and engagement* in the patient with online multifaceted feedback (Irazoki et al., 2020; Wilson et al., 2022). A meta-analysis and comprehensive review of the research in this area revealed that older adults are highly satisfied related to the usage of tablets and other technology devices (Ramprasad et al., 2019). In particular, these people reported higher satisfaction for variables such as the availability, perceived helpfulness, online feedback on completion rates and usability of the technologies themselves. Indeed, technology allows for greater *flexibility in modulating stimuli* and provides *immediate and contextualized feedback* to the patient (Lorenz et al., 2019; Irazoki et al., 2020; Pappadà et al., 2021; Wilson et al., 2022).

Secondly, these novel interventions through technological devices enable reaching people at their homes, reducing costs and time, thus minimizing *environmental constraints* (Burton and O'Connell, 2018; Ge et al., 2018; Irazoki et al., 2020).

Moreover, in terms of *effectiveness, validity, reliability, and patient satisfaction*, there is early evidence that telemedicine and technological CT interventions are comparable to traditional face-to-face procedures (Ge et al., 2018; Georgopoulou et al., 2023). Again, Ramprasad et al. (2019) found no evidence of actual improvement in clinical or behavioral variables in patients using technology-based cognitive rehabilitation. Despite that a lot has been published on the advantages and disadvantages of different forms of rehabilitation (Bernini et al., 2023a,b; Mantovani et al., 2020), there is still an urgent necessity of further studies aiming to specifically compare standard versus technological CT. Tools and procedures of clinical and research rehabilitation protocols—both paper-pencil and technological—are not always clear and well-detailed, so preventing the reproducibility and, in turn, the scientific evaluation of these approaches (Ge et al., 2018; Sandoval-Lentisco et al., 2024). The detailed description of the procedure for creating and choosing technological items is pivotal to analyze possible involuntary confounding variables in the transition from interventions based on paper-pencil or computerized stimuli. Thus, to date, the lack of a reasonable amount of well conducted randomized controlled trials and longitudinal studies makes it difficult and incautious to draw firm conclusions (Ge et al., 2018; Mantovani et al., 2020; Maresca et al., 2020; Kang et al., 2022).

Regarding this point, the great variability of technological CT interventions may be partly responsible for the challenges in evaluating the efficacy of these treatments. Indeed, there are several types of technology-based cognitive rehabilitation approaches that are relevant discussing at this stage. The first is *computer-based cognitive technology*, which includes cognitive games that may be played on computers or mobile devices like tablets and smartphones (Irazoki et al., 2020; Wilson et al., 2022). The second one is *virtual reality* (VR), which is a computer simulation of a man-made setting where patients can engage in real time using both visual and non-visual modalities (such realistic noises). VR systems can be categorized by being *non-immersive* (monitor with a two-dimensional virtual environment and interaction via a controller like a mouse or joystick) or *immersive* (three-dimensional virtual world). Considering the two different VR modalities, the immersive approach offers a greater cognitive and motor challenge (Leung et al., 2022). At the same time, it is appropriate to note that, more generally, individuals with cognitive decline may experience confusion, exhibiting difficulty or inability to distinguish between reality and VR, or, conversely, developing dependence on excessive VR use (Leung et al., 2022).

Finaly, not much is known on *why* and *how* to pass from a standard approach based on paper-pencil to a technological one (Ge et al., 2018; Gates et al., 2019). Rarely, one solution fits all. Literature showed that technological interventions enable the rehabilitation of single- or multiple- domain(s), increasing the monitoring and control (Ge et al., 2018; Irazoki et al., 2020; Georgopoulou et al., 2023; Kang et al., 2022). The issue might be that, in some cases, a conventional approach may be less effective than a technological one in the rehabilitation of single specific domains, or vice versa. Indeed, the effectiveness of CT is linked to multiple variables of the patients' characteristics and their environment, so that the combination of these elements can function as facilitators or hindrances depending on the situation. For instance, relevant differences can be determined by environmental factors such as facility accessibility and related costs. For a patient living in a rural area, a technological CT can be more effective as it enables remote use and monitoring (Ramprasad et al., 2019; Irazoki et al., 2020). Similarly, technological CT could be appreciated by patients with physical disabilities who cannot undergo a face-to-face training section due to difficulties in reaching the clinic or by individuals with sensory deficits that technology can overcome (e.g., stronger visual and auditory feedback). In this sense, technological CT allows for full customization of treatment based on the individual's needs (Irazoki et al., 2020). On the contrary, some patients may be demotivated and lack adherence to technological CT, as they are not used to adopting technological devices such as computers or other smart devices, nor are they supported by a family caregiver (Irazoki et al., 2020; Grigorovich et al., 2021). In this case, lifestyle habits and sociodemographic factors may play a role in the preference of standard vs. technological approaches for CT, from both a clinical perspective and in terms of the patient's user experience (González-Fraile et al., 2021; Bernini et al., 2023a).

### 3.2 The efficacy of cognitive training: it is all a matter of time

As far as we know, regardless of the standard or technological approach, there is currently no agreement on the duration of CT to be effective in both the short and long term. Specifically, there is heterogeneity in duration and frequency of CT, respectively ranging from 4 to 26 weeks, and from two to three times per week for <1 h (Irazoki et al., 2020; Contreras-Somoza et al., 2021). While considering the need to make different treatment protocols comparable in order to study their effectiveness, the fact that modern medicine is becoming more and more patient-oriented should not be ignored. In fact, it clearly appears that developing individualized treatment plans that are tailored to each patient's unique requirements may be advantageous. Thus, it is crucial in clinical practice to avoid standardizing frequency and duration of treatment. Conversely, it is recommended to calibrate these aspects through a constant discussion between the healthcare professionals involved in the treatment and a regular reassessment of the patient's individual therapeutic goals, taking into account both short- and long-term objectives (Fleeman et al., 2015).

Even without a clear guideline, it seems that CT must be frequent and prolonged to be effective (Mantovani et al., 2020; Bahar-Fuchs et al., 2013). From a broader perspective, CT can be part of an active lifestyle that is a protective factor against dementia (Ownby and Waldrop, 2023). However, literature and clinical practice suggest that cognitive impairment can affect non-adherence, in particular the unintentional one caused by forgetfulness or cognitive deficits that prevent the correct implementation of therapies (Bahar-Fuchs et al., 2013; Dequanter et al., 2022; Nahas et al., 2024). Besides this, it is necessary that the patient is fully engaged in the CT, that should be considered a pivotal non-pharmacological intervention for preventing cognitive decline and maintaining cognitive resources (Dequanter et al., 2022). Indeed, modern conceptualization of adherence is intended as a complex and broader process referring to “*the extent to which a person's behaviour-taking medication, following a diet, and/or executing lifestyle changes, corresponds with agreed recommendations from a healthcare provider*” (Sabaté, 2003, p. 3). That is, independently from the approach—standard or technological—the success of the intervention is likewise given by the patient's adherence in terms of persistence and correct implementation of the proposed CT (El-Saifi et al., 2018). Thus, it is essential to identify all potential obstacles and enablers for adherence in order to stay persistent over time.

For instance, interventions based on telehealth home monitoring may improve adherence in the elderly because they solve the problem of travel to reach healthcare facilities (El-Saifi et al., 2018). The literature recommends also the adoption of a patient-centered approach, in which CT is a shared decision (Ranzini et al., 2020). In their study, Fleeman et al. (2015) proposed an interdisciplinary approach to cognitive rehabilitation known as the Integrative Cognitive Rehabilitation Programme Theoretical Model (ICRP) that takes its cue from the theory of Distributed Cognition. The latter conceives cognition as a socio-technical system in which individuals, objects, processes and contexts interact in such a way that, from a rehabilitation perspective, cognitive support technology and formal and informal caregivers act as compensatory tools to improve cognitive functioning in the individual's environment (Hutchins, 1995). Consequently, the ICRP is based on the idea that the integration of compensatory rehabilitation strategies, which consider the uniqueness of the individual according to the biopsychosocial model and the Distributed Cognition theory, allows for the development of a rehabilitation plan that maximizes the individual's potential, thus facilitating the personalization of the intervention for each patient (Fleeman et al., 2015). In this way, the already patient-centered treatment can benefit from the specific competences of a multidisciplinary team, favoring a continuous exchange and comparison between the different professionals involved.

Moreover, sociodemographic and lifestyle habits may push the patient to prefer and, in turn, to easily adhere to, standard or technological CT (Kerkhof et al., 2022; Bernini et al., 2023a). These aspects should be carefully considered as they may help foster the patient's engagement and motivation. For example, today's older individuals may have discomfort with unfamiliar technological devices, but this condition may reverse in tomorrow's seniors who may instead be less accustomed to paper and pencil (Garcia Reyes et al., 2023). It is essential to consider and implement supportive factors in the rehabilitation process that can enhance the perceived effectiveness of CT, such as external support, the therapist-patient relationship, satisfaction, and self-perceived efficacy regarding the intervention. These elements may improve patient motivation and the usability experience of the tools, ultimately supporting adherence to rehabilitation and generalizing the skills acquired (Nahas et al., 2024).

Lastly, we have to consider cognitive impairment as a *family* condition. Consequently, it is crucial for adherence to pay attention to the patient-caregiver dyad (Giardini et al., 2018; Torlaschi et al., 2022). Indeed, CT should be supportive without being a further burden for the caregiver (Giardini et al., 2018). Moreover, the caregiver role may be played by different people, such as wives or husbands, sons or daughters, as well as homecare assistants (Bremer et al., 2017). Considering everyone is a child of his time, choosing the more functional approach depends also on the caregivers' characteristics and, in turn, on the patient-caregiver dyad needs (Cunnah et al., 2021).

## 4 Open questions, open opportunities

Bearing in mind what we discussed above, the scientific community cannot approximately arrange technological CT—galvanized by artificial intelligence and new technologies. Instead, it must operationalize, standardize and clarify the creation of technological stimuli delivered through technological devices on the basis of the know-how inherited by paper-pencil stimulation.

Concerning the transition from standard to technological CT, future research must first address the following central issue: are there significant differences in therapeutic outcomes between traditional rehabilitation practices and those integrating new technologies? (Ramprasad et al., 2019). At present, it is not possible to provide clear indications, but it is essential to initiate studies that can answer it. To this end, it would be useful to conduct well-structured research that shows whether patients actually improve, as measured by objective clinical and behavioral variables. Furthermore, it would be crucial to involve larger and more heterogeneous patient samples, considering minority populations in particular, especially those with low socioeconomic status, residing in rural areas, or receiving home care (Ramprasad et al., 2019). In this regard, it would be desirable to carry out in-depth studies that promote cognitive therapy pathways using telemedicine to address the need to include minorities (Dissanayaka et al., 2023).

Moreover, neuroimaging studies should corroborate the behavioral evidence and understand the neural mechanisms underlying technology-based cognitive interventions. Regarding this, Leung et al. (2022) proposed interesting insights, specifically mentioning the possibility of conducting technology-based cognitive interventions alongside non-invasive brain stimulation, such as transcranial magnetic stimulation (TMS) or transcranial direct current stimulation (tDCS). It would thus be possible to understand whether such stimulation tools can maximize the benefits of CT and, more generally, to investigate the effects of such therapies in older adults with physical and/or cognitive challenges (such as, for example, wheelchair patients who are unable to perform upper and lower body movements during VR therapies) (Leung et al., 2022).

If valuable, the transition from standard to technological CT has to take into account the challenges that some today's elderly patients suffering from cognitive impairment may face when using electronic devices. Therefore, from a practical standpoint, it would be beneficial to promote more effective social support, as engaged caregiver or professionals, which can help over time the patient using technology, thus increasing levels of self-efficacy and motivation, as well as maximizing potential benefits (Leone et al., 2018; Nahas et al., 2024). It would be also important to improve the perceived usability of these technologies by adopting user-centered designs. As previously discussed, this approach could promote treatment adherence and help overcome some of the limitations associated with the complexity of some technologies (Grigorovich et al., 2021).

Finally, from a broader perspective, we have to remember that maybe it is not only a matter of *how* but also of *how long*: cognitive stimulation—standard or technological—should perdure over time in the day-to-day life to lead to a winning and preventive lifestyle. In the present aging era, fostering the patient adherence toward CT and active living is another core mission for the healthcare community.

Regarding this, it is essential not to overlook the role of the professionals and healthcare teamwork. In fact, beyond the rehabilitation approach used, the quality of the relationship between patient-therapist and between the different professionals plays a crucial role in patient engagement and in the perceived quality of the proposed rehabilitation pathway, influencing adherence and, potentially, outcomes (Nahas et al., 2024). In order for practitioners to be able to act at their best, it is essential that clear guidelines are drawn up, which constitutes yet another challenge to be met by those working in this field (Nahas et al., 2024).

## 5 Conclusion

Thinking about these issues may help to broaden clinical and research horizons and perspectives. It is important to fully comprehend what we are doing, controlling as much as possible the potential benefits and pitfalls of new CT approaches compared to standard paper-pencil ones, in order to decide which direction to go in and what is the true and realistic purpose (Kerkhof et al., 2022). Perhaps the healthcare community should consider how to use CT at its best: to enable the individual living as best as they can? To counterattack the cognitive decline? Or to patch up the deficit? Does this mean that we should finally rehabilitate the person, the disease or the deficit? In summary, the questions deserving further discussion are: how can we switch to novel technological interventions maximizing advantages without losing the know-how gained by the decades of paper-pencil CT? How can we be flexible and effective in choosing to use one intervention rather than another one? We cannot yet provide solid answers due to the heterogeneity of experiences (Ge et al., 2018; Sandoval-Lentisco et al., 2024; Ramprasad et al., 2019), but we can reflect and share ideas and discussion on the complexity and open gaps in CT. Finally, what can we save from the past? What should we change in the future?
